# Comments on Proteomic Investigations of Two Pakistani *Naja* Snake Venoms Species Unravel the Venom Complexity, Posttranslational Modifications, and Presence of Extracellular Vesicles. *Toxins* 2020, *12*, 669

**DOI:** 10.3390/toxins12120780

**Published:** 2020-12-08

**Authors:** Theo Tasoulis, Tara L. Pukala, Geoffrey K. Isbister

**Affiliations:** 1 Clinical Toxicology Research Group, University of Newcastle, Callaghan, NSW 2308, Australia; theo.tasoulis@newcastle.edu.au; 2 School of Physical Sciences, University of Adelaide, Adelaide, SA 5005, Australia; tara.pukala@adelaide.edu.au

We read with interest the article by Manuwar et al. [1] on the proteomics of the venoms of two Pakistani *Naja* spp. (Cobras; *Naja naja* and *Naja oxiana*). This study identified the different toxins in two *Naja* venoms, classifying these toxins into known protein families. Although this may provide some useful information on the diversity of toxins in the venom, it does not provide information on the abundance of each protein family in the venoms of the snakes, as purported by Manuwar et al. The authors incorrectly equated the number of different toxins in each protein family to the abundance of each toxin/protein family in the venom. They also confuse peptide fragments (Table 1 in ref. [1]) with proteins (Tables 2 and 3 in ref. [1]), making an incorrect assessment of diversity. There is an important distinction between abundance and diversity, because the toxicity of a venom reflects the amount or abundance of a particular protein family (e.g., Three-finger toxin (3FTx)) and not the number of different 3FTxs there are in the venom (diversity).

Relative toxin abundance calculations are always an approximation and it is difficult to achieve complete accuracy. The most common method used to determine toxin abundance is to fractionate the venom chromatographically, run each fraction on a separate gel lane using Sodium Dodecyl Sulfate Polyacrylamide Gel Electrophoresis (SDS-PAGE), identify the bands in the gel lanes (using mass spectrometry), perform densitometry on the gel (if more than one band per lane), and then measure the area under the chromatogram curve. This provides a correct estimate of the proportion of the venom made up by each of the protein families [2,3,4].

In contrast to this, the authors’ methods state that they counted the number of different toxins in each protein family and divided this by the total number of toxins in the venoms. However, the authors called this process “abundance.” Based on this definition and the authors identifying 22 different 3FTxs in the *N. oxiana* venom (Table 3 and Figure 3 in ref. [1]), 3FTxs made up 22/140 (16%) of the venom. Similarly, 3FTxs would therefore make up 54/365 (15%) of *N. naja* venom (Table 2 in ref. [1]). What is more confusing is that they, in fact, calculated the abundance from Table 1 as the number of peptide fragments created by trypsin digestion for each protein family divided by the total number of fragments. They therefore incorrectly suggested that this was the abundance of 3FTxs, where, in fact, this was the number of different 3FTx fragments in the venom.

Manuwar et al. then compared the percentage abundances from their study with the true abundance of each protein family in *N. naja* from India [5,6], and suggested that *Naja* spp. from Pakistan express quite different venom proteomes. However, the venom proteome of *N. naja* from Pakistan has already been published by Wong et al. [2], who showed that the venom consists of 75% 3FTx and not the 21% claimed by Manuwar or the 15% that we recalculated when using their methods. Compared to a more recently published venom proteome of *N. naja* from western India [5], Pakistani *N. naja* do possess a different venom proteome, but in the opposite way. The Pakistani populations instead express higher levels of 3FTx (75%) compared to 51% in western Indian *N. naja*. Previously published venom proteomes of 11 species of *Naja* found that the abundance of 3FTxs in *Naja* venom proteomes was 56% to 84% of the total venom (depending on the species) [7].

The difference between toxin abundance and toxin diversity is central to understanding differential venom toxicity and its biological and medical implications. The difference between the two is clearly demonstrated in many venoms, in which one protein family makes up most of the venom but there are only a few different toxins in that protein family. For example, the Malayan blue coral snake (*Calliophis bivirgata flaviceps*) possesses 23 different proteins in its venom proteome [8]. However, just one toxin (cytotoxin maticotoxin) makes up 23% of the whole venom, while nine different snake venom metalloproteases (SVMP) make up 19% of the whole venom. Using the erroneous method of Manuwar, we would instead estimate the abundance of maticotoxin as 1/23 or 4% and of SVMP as 9/23 or 39%. Another example is the sea krait *Laticauda colubrina* [9]. The venom proteome was found to be composed of 31 toxins of which nine were 3FTxs, which would be an abundance of 29%. However, the abundance was, in fact, estimated to be 66% of the total venom using appropriate methods.

We recommend that authors publishing results on venom proteomes need to clearly state, both in the text and in the figure captions, what their results actually represent. For ease of interpretation, it would be preferable if each sector on the pie chart gives the percentage of total venom (of each protein family) followed by the number of different toxins in brackets. The words “amounts” and “abundance” cannot be used interchangeably with “diversity” and “complexity” when they are two different things. This will only lead to confusion and incorrect conclusions when making geographic comparisons, or worse, conclusions on potential venom toxicity and antivenom production (as Manuwar et al. have done) with medical implications.
